# Reinvent 4: Modern AI–driven generative molecule design

**DOI:** 10.1186/s13321-024-00812-5

**Published:** 2024-02-21

**Authors:** Hannes H. Loeffler, Jiazhen He, Alessandro Tibo, Jon Paul Janet, Alexey Voronov, Lewis H. Mervin, Ola Engkvist

**Affiliations:** 1 Molecular AI, Discovery Sciences, R&D, AstraZeneca, Gothenburg, Sweden; 2 Molecular AI, Discovery Sciences, R&D, AstraZeneca, Cambridge, UK

**Keywords:** Generative AI, Reinforcement learning, Transfer learning, Multi parameter optimization, Recurrent neural networks, Transformers

## Abstract

REINVENT 4 is a modern open-source generative AI framework for the design of small molecules. The software utilizes recurrent neural networks and transformer architectures to drive molecule generation. These generators are seamlessly embedded within the general machine learning optimization algorithms, transfer learning, reinforcement learning and curriculum learning. REINVENT 4 enables and facilitates de novo design, R-group replacement, library design, linker design, scaffold hopping and molecule optimization. This contribution gives an overview of the software and describes its design. Algorithms and their applications are discussed in detail. REINVENT 4 is a command line tool which reads a user configuration in either TOML or JSON format. The aim of this release is to provide reference implementations for some of the most common algorithms in AI based molecule generation. An additional goal with the release is to create a framework for education and future innovation in AI based molecular design. The software is available from https://github.com/MolecularAI/REINVENT4 and released under the permissive Apache 2.0 license. **Scientific contribution**. The software provides an open–source reference implementation for generative molecular design where the software is also being used in production to support in–house drug discovery projects. The publication of the most common machine learning algorithms in one code and full documentation thereof will increase transparency of AI and foster innovation, collaboration and education.

**Supplementary Information:**

The online version contains supplementary material available at 10.1186/s13321-024-00812-5.

## Introduction

Molecular Design is the creation of novel molecules with desired properties for a given problem in chemistry, material science or nanotechnology. Ideally, this would be done in a systematic fashion rather than through trial–and–error. In drug discovery this is often approached with rational drug design [1] which makes significant use of computers and algorithms to generate novel molecules. Specifically, so–called *de novo* methods create molecules from scratch i.e. without or little prior molecular information [2]. In this context we will discuss *de novo* molecular design using generative AI models [3] and focus in particular on the implementation of the REINVENT software. The application of AI in drug discovery has been debated and challenged. It is therefore of high value to the scientific community that there exist reference implementations in the public domain of the most common algorithms for generative molecular design to facilitate a nuanced debate. It is also hoped that the released software can contribute to the education and innovation in the field of AI-based molecular design.

Generative AI models capture the underlying probability distribution of known molecules and their local relationships to each other (distribution learning). This distribution is in principal unknown and thus the modelled distribution only an approximation. However, we can define a “chemical space” in this way from which can be extrapolate into novel chemical space. Statistical methods are then used to sample from the distribution i.e. generate novel molecules. The field is still relatively new and experimental validations in the public domain are slowly starting to emerge [4–7] but various useful perspectives and reviews of the methodology have already appeared in the literature [3, 8–11]. Here, we will focus on small molecule design but other modalities are being investigated as well [12, 13].

Numerous AI model architectures have been developed for generative molecular design e.g. variational autoencoders (VAE) [14, 15], generative adversarial networks(GAN) [16], recurrent neural networks (RNN) [6, 17–20], transformers [21, 22], flow models [23, 24] and diffusion models [25] (either directly generating in 3D [26, 27] or from 1D SMILES strings [28]), reaction based models [29]. The molecular representation used for these algorithms can be different and can be typically categorized with their dimensionality [13, 30]. All these methods have their relative merits and there is no one solution that uniformly outperforms the others. Various benchmarks have been designed to validate technical aspects of molecular generation and optimization [31, 32].

Molecular design can be framed as an inverse design problem. In forward design we would modify existing compounds until they satisfy our criteria while inverse design first states the properties the molecule must possess and thus informs an algorithm on how to create the molecules. Drug molecules in particular must follow a stringent property profile before being approved as safe and efficacious medicines including affinity to the target(s), selectivity against off–targets, the right physico–chemical properties, the right ADME (absorption, distribution, metabolism, excretion) characteristics, good PK/PD (pharmacokinetics/pharmacodynamics), favourable toxicology, chemical stability. Also very importantly synthesizability [33], the potential to scale-up a synthetic route and the requirements of green chemistry [34]. This highlights the complexities in designing a successful drug and the requirements for algorithms to solve this. The inverse design problem is the attempt to map a (manageable) number of properties back to a vast chemical space. Various attempts have been made at predicting the success of a compound in the clinical stages by trying to find the “right” combination of molecular properties [35, 36].

Molecular design should be seen as part of the DMTA (design, make, tests, analyse) cycle. Generative models can contribute to the design part while robotic systems can contribute to make, test and analyse in an attempt to create a fully automated closed–loop experimentation system [37, 38]. The ambition is to speed–up molecular design in a systematic and efficient manner. Levels of automation have been defined and it is clear that decision making and synthesizability are key factors in achieving full automation [39].

In this contribution the progress of REINVENT as a framework for molecular generative AI is described. REINVENT is in production and continuously maintained. REINVENT tackles the inverse design problem through reinforcement learning [19, 20, 40–42] using RNNs and transformers as deep learning architectures based on SMILES strings as molecular representation. Here we describe the new version 4 emphasizing novel features like combined reinforcement/curriculum learning (RL/CL) staged learning, new transformer models for molecule optimization, full integration of all generators within all algorithmic frameworks: transfer learning (TL), RL, CL, reworked scoring subsystem utilizing a plugin mechanism for easy extension and the TOML configuration file format in addition to JSON (incompatible with previous releases). REINVENT 4 is a well–designed and complete molecular design software solution. The code base has been largely rewritten and all software and models are available in a single repository. The descriptions of the original REINVENT version 1 and version 2.0 have been published elsewhere [19, 20]. The code of version 3 has been released as open–source software but without accompanying manuscript.

REINVENT has been shown to outperform many other methods of molecular optimization in terms of sample efficiency [43] but is also successful in proposing realistic 3D molecules as shown in a recent docking benchmark for generative models surpassing many graph–based methods [44]. It has also been demonstrated that the algorithm can produce chemistry outside of the training set with certain CL protocols [45]. Table 1 compares functionalities in REINVENT 4 with the previous version 2.0 and DrugEx version 3 [18]. DrugEx is another open–source generative AI software similar in spirit to REINVENT and also in production state. We do not compare here to research based software released for in a specific publication for reproducing the claims in the publication.

**Table 1:** Comparison of major functionalities in REINVENT and DrugEx

Functionality	REINVENT 4	REINVENT 2.0 [20]	DrugExV3 [18]
*De novo* design	$\checkmark$	$\checkmark$	$\checkmark$
Scaffold design	$\checkmark$		$\checkmark$
Linker design	$\checkmark$		
Molecule optimization	$\checkmark$		
Reinforcement Learning	$\checkmark$	$\checkmark$	$\checkmark$
Curricululm Learning	$\checkmark$		
Transfer Learning	$\checkmark$	$\checkmark$	$\checkmark$
Input format(s)	TOML/JSON	JSON	command line

## Theory

Here the theory underlying REINVENT 4 is described. The specifics of the software is also highlighted. A comprehensive collection of various capabilities that are otherwise distributed in previous publications are provided [19, 20, 46–48].

### Generating molecules

All REINVENT 4 models consist of sequence–based neural network models that are parameterized to capture the probability of generating tokens *t* in an auto-regressive manner. The models are called agents. A sequence describes a SMILES string which represents a molecule. The tokens are characters or character combinations in SMILES strings, see Affitional file 1. Tokens are drawn from a fixed vocabulary $t\in V$, created at training time (and fixed at inference time implying that input SMILES must follow the model’s vocabulary). A special termination token indicates completion of the sequence. REINVENT 4 supports unconditional and conditional agents which describe probability distributions over sequences from *V*. The joint probability $\textbf{P}(T)$ for unconditional agents of generating a particular sequence *T* of length $\ell$ with tokens $t_1, t_2,\ldots , t_{\ell }$ is given by

1 $$\begin{aligned} \textbf{P} (T) = \prod _{i=1}^{\ell }\textbf{P}\left( t_i\vert t_{i-1}, t_{i-2},\ldots , t_1\right) . \end{aligned}$$

Conditional agents model a joint probability $\textbf{P}(T\vert S)$ of generating a particular sequence *T* of length $\ell$ given an input sequence *S* given by

2 $$\begin{aligned} \textbf{P} (T\vert S) = \prod _{i=1}^{\ell }\textbf{P}\left( t_i\vert t_{i-1}, t_{i-2},\ldots , t_1, S\right) . \end{aligned}$$

From Eqs. 1 and 2 we define the negative log-likelihood as

3 $$\begin{aligned} NLL(T)= & {} -\log \textbf{P} (T) = -\sum _{i=1}^{\ell }\log \textbf{P}\left( t_i\vert t_{i-1}, t_{i-2},\ldots , t_1\right) \end{aligned}$$

4 $$\begin{aligned} NLL(T \vert S)= & {} -\log \textbf{P} (T \vert S) = -\sum _{i=1}^{\ell }\log \textbf{P}\left( t_i\vert t_{i-1}, t_{i-2},\ldots , t_1, S\right) \end{aligned}$$

for $\textbf{P} (T)$ and $\textbf{P} (T\vert S)$, respectively.

As in previous versions, a number of *prior* agents are made available (details in Priors). These are foundation models, trained in an unsupervised fashion with teacher–forcing [49] using SMILES strings from large public data sets of molecules. The teacher–forcing strategy feeds the model with the actual output from the data set (ground truth) as input during training instead of the network’s generated output. Once trained, REINVENT 4 agents acquire an understanding of the syntax of the SMILES strings, enabling them to generate valid molecules. In practice this amounts to updating the weights of the models to decrease the negative log-likelihood of either Eqs. 3 or 4 (depending on the model type) over all molecules in the training data set.

Because the models are trained on all input molecules in the same way, priors represent unbiased molecule generators (however, still biased due to the limited chemical space of the training set), resulting in a theoretically uniform distribution over the training molecules. These models possess the capability to sample molecules that goes beyond just re-sampling the training data. For example, a prior trained on 1 million molecules can easily sample 100 s of millions of unique, valid molecules [50]. Training priors on multiple equivalent SMILES representations of the same molecule has been shown to result in more expressive priors [51].

REINVENT 4 supports two decoding strategies, namely multinomial sampling used for e.g. by [22, 52] and beam search [53]. Multinomial sampling allows fast, non–deterministic generation of compounds. At each step, a token is randomly selected based on the probability distribution over the vocabulary. The current implementation supports a positive temperature–like parameter *K* (default $K=1$) used to scale the probability distribution. When decreasing the temperature, i.e. $K < 1$, the distribution becomes sharper: the chance of high probability tokens being selected increases, conversely the chance of low probability tokens being selected decreases. This results in less randomness and so more determinism. More randomness is introduced when the temperature is increased ($K > 1$) which causes the distribution to become flatter and lower probability tokens to be selected more preferentially. Multinomial sampling might suffer from mode collapse i.e. sampling might tend to produce a small number of compounds. The computational complexity for multinomial sampling is $O(\ell \cdot |V|)$, where $\ell$ is the length of tokens and |*V*| is the size of the vocabulary.

In contrast, beam search is a deterministic approach that always generates unique compounds. However, it is computationally more expensive than multinomial sampling as it scales as ${\mathcal {O}}(B \cdot \ell \cdot |V|)$, where *B* is the beam size. Note that for both techniques the complexity of the underlying generative model impacts the performance. This complexity arises because SMILES strings are generated iteratively by feeding the transformer with $n-1$ tokens to obtain the *n*th token. In fact, for multinomial sampling, the model needs to compute the probabilities of each possible token, while for beam search, we also need to store the *B* most probable SMILES subsequences.

REINVENT 4 includes Mol2Mol, a conditional prior agent, as described in [54] which allows for a systematic exploration of the chemical space. The prior was trained on over 200 billion pairs from Pubchem [55] for which their Tanimoto similarity, calculated with ECFP4 count fingerprints, was $\ge$ 0.50. Furthermore, the prior training was regularized with the ranking loss, allowing to directly link negative log-likelihood to similarity.

### Transfer learning

Transfer Learning (TL) are methods that re–use existing knowledge to facilitate the learning of another, related task. In machine learning this is typically applied to retrain a large model with a small amount of data to efficiently obtain a new improved model and can accordingly be used when only little data is available for the new task. TL can thus be seen as fine–tuning an existing model. TL has been applied successfully in drug discovery [56] specifically it has been shown that a focused generative model can produce a similar fraction of active molecules as experience replay [7] (see "Inception" for an explanation of experience replay).

In REINVENT 4, transfer learning is conceptualized as retraining of a prior model using the same teacher–forcing strategy as in the pre–training of the prior model (see Generating Molecules). A small, task–focused data set is chosen, for example a data set containing active molecules for a particular drug target. TL then creates a new agent that is specifically biased toward generating analogues to these active molecules. In this way the agent will be able to generate relevant molecules more quickly.

### Reinforcement learning

Reinforcement Learning (RL) describes various optimization methods in machine learning where an agent acts in an environment to learn a strategy (policy or goal). The agent is rewarded when the action is beneficial to the goal or receives negative feedback when the action isn’t beneficial. For example, in generative molecular design the goal is to drive a prior model such that the generated molecules satisfies a predefined property profile. RL is a frequently used optimization method in drug discovery [56].

**Fig. 1:**
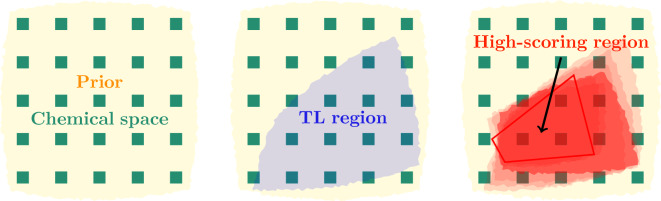
Illustration of idealized behavior of priors, transfer learning agents and reinforcement/staged learning agents. In all cases, the models describe the probability of sampling a given token sequence corresponding to a specific molecule (green squares), represented by a colored fill. The prior model is trained to increase probability over all drug–like molecules. A transfer learning agent built from this prior increases the likelihood on a specific region (blue, middle). In staged learning (red, right), starting from the transfer learning agent, likelihood of sampling high-scoring sequences is iteratively increased, resulting in concentration on high-scoring regions (red polygon)

In REINVENT, RL is used to iteratively bias the molecules generated by an agent (normally a prior or transfer learning agent) via a policy gradient scheme (Fig. 1). In a drug discovery project, the aim is typically not to create a new model but rather to generate molecules which score highly according to the provided scoring function. This is achieved by providing a scalar score, $S\in [0,1]$, for each token sequence *T* (representing a molecule) generated in each epoch. This is used to define a so–called augmented likelihood for each sequence as

5 $$\begin{aligned} \log \textbf{P}_\text {aug} (T) = \log \textbf{P}_\text {prior} (T) + \sigma \textbf{S} (T) \end{aligned}$$

First proposed in [19], this expression combines the reward signal with the likelihood of the sequence under the fixed, generalist prior model, which serves as a regularization term to control the generation of plausible sequences from a chemistry viewpoint. The balance between the reward and regularization is controlled with the scalar parameter $\sigma \ge 0$. $\sigma$ is a user–adjustable parameter and it can have a major impact on performance [43].

In the “Difference between Augmented and Posterior” (DAP) strategy [46], the augmented likelihood is used to define a loss for each sequence in the batch, computed as

6 $$\begin{aligned} {\mathcal {L}} (T) = \left( \log \textbf{P}_\text {aug} (T) - \log \textbf{P}_\text {agent} (T)\right) ^2 \end{aligned}$$

were $\log \textbf{P}_\text {agent} (T)$ is the likelihood of sequence (*T*) under the current agent. This loss is averaged over all molecules generated in a batch and then the current agent is updated to reduce this loss via a stochastic gradient descent method (Sect. "Other Parameters"), i.e. bring the likelihood of the sequences closer to $\log \textbf{P}_\text {aug} (T)$. The only term in 6 that is a function network parameter is $\textbf{P}_\text {agent}$. The presence of the prior in these expressions constrains how far the RL agent can stray from the prior, similar to proximal policy gradient methods, except that the prior is static during the RL process.

This definition of the augmented likelihood and loss function has a few non–obvious implications. Firstly, the form of Eq. 5 and non–negativity of the score means that the likelihood for sequences is only increased (or unchanged) relative to the prior in each epoch. A molecule that obtains a zero score will have a augmented likelihood identical to that obtained under the prior model, and so low–scoring molecules have little impact on the state of the agent, i.e. there is limited learning from negative examples early in the run.

**Fig. 2:**
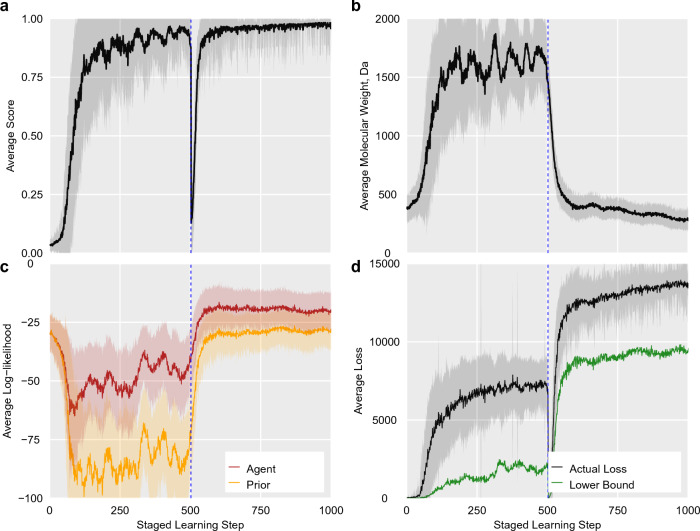
Simple experiment demonstrating adaptable learning behavior starting with the default REINVENT 4 agent. 500 epochs of RL are run with a scoring function that rewards molecular weight $\ge 1500$ Da, before it is switched in a second stage that rewards molecular weight $\le 1500$ Da, showing the score **a**, molecular weight **b**, agent and prior likelihoods **c** and loss function **d** averaged over all molecules at the end of each epoch. The loss lower bound (Eq. 7) is also shown in **d**. A dashed line indicates the change of scoring function. The run used default settings: batch size of 128 and $\sigma =128$

However, the behavior can be markedly different in the case of dynamic variation in how the reward is computed during the run. To illustrate this, we consider a simple experiment where we start with the REINVENT 4 prior: we run 500 epochs of RL with standard settings ($\sigma =128$) and a scoring function that encourages generation of extremely large molecules (1500 Da) relative to the drug–like molecules in the prior. At the 500 epoch mark, we move to a second stage where the scoring transform is reversed, encouraging the generation of molecules with $\le 500$ Da molecular weight. The agent is rapidly able to solve both tasks (Fig. 2a and b).

Despite learning to make large molecules that are highly unlikely under the prior ($\log \textbf{P}_\text {prior} (T)< -75$, Fig. 2c) for 500 epochs, the agent is rapidly able to adapt to the change in scoring function and generate small molecules again by epoch 550. This transition period is accompanied by the agent likelihood regressing back to be similar to the prior likelihood, before separating again. This plasticity is a capacity that makes these systems adaptive in various settings, for example active- [57] or curriculum learning [58] settings.

Since Eq. 5 have values in $\left( -\infty , \sigma \right]$, $\log \textbf{P}_\text {aug}$ is not guaranteed to be an obtainable log-likelihood for the discrete distribution of sequences that can be generated by these models (i.e. $\le 0$), particularly for high scoring sequences and large values of $\sigma$. This is not a problem in practice, and while various other loss functions have been considered [46] (and remain available, see RL Learning Strategy), DAP typically provides the most rapid learning and serves as a robust general purpose method. The combination of Eqs. 5 and 6 means that the loss for any sequence is lower-bounded by

7 $$\begin{aligned} \begin{aligned} {\mathcal {L}} (T)&\ge \max (0, \log \textbf{P}_\text {prior} (T) + \sigma \textbf{S} (T))^2 \end{aligned} \end{aligned}$$

This, combined with the observation that the loss is computed with respect to a new batch of ideas for each epoch, can lead to counter-intuitive behaviour where the loss function can increase during RL as the score increases (Fig. 2d). However, this is the expected behaviour as the loss is reduced on the previous batch of molecules, which are not re–evaluated but the agent in the next epoch. Generally, the loss lower bound is highest for high scoring batches that are also likely under the prior, as in the case of the molecules with drug-like molecular weight generated in the second part of the experiment.

## Methods

### Reinforcement learning

Reinforcement Learning (RL) is the main molecule optimization method in REINVENT. RL has been re–framed in the new version into staged learning (see "Methods") which allows multiple successive and consecutive RL runs with varying parameters.

Each stage will write out a CSV file which contains all information about negative log likelihoods, total and individual component scores and the sampled SMILES strings. The CSV file is created in real time i.e. every RL epoch is immediately written to disk (the operating system may impose buffering such that the file is written in chunks). This implies that the data in the CSV is unfiltered meaning that invalid SMILES and low scoring compounds will be logged also. It is the user’s responsibility to post–process this file in a meaningful way.

#### RL learning strategy

Previously, four different RL learning strategies in REINVENT were described [46]. It was found that DAP displayed the best learning rate while the others showed very little or no improvement. In version 4 we still offer all four functions but we recommend the DAP for practical use. The other three are still available but are deprecated meaning that they might be removed in future releases.

#### Diversity filter

The diversity filter is, as its name suggests, a mechanism to promote molecular diversity during an RL run. This is primarily based on scaffold diversity using a memory with a user adjustable size. The memory is organized into “buckets” which hold a given scaffold. When the bucket is full every further occurrence of that scaffold enforces a zero score for the whole molecule. Scaffolds can be computed as Murcko type scaffolds, “topologically” which means the scaffold is determined disregarding elements and bond types (unlabelled graph) and scaffold similarity which stores the most similar scaffolds found so far. In the current implementation all scaffold filters also contain a global SMILES string memory of size 1. This means that every further occurrence of the same canonical SMILES string is scored with zero. This happens both locally i.e. within a batch and globally i.e. over the whole run. This implies that SMILES are *not* de–duplicated in advance conforming with previous versions. Otherwise the behaviour would be altered, see Eq. 6 where duplicates have zero score but their negative log likelihoods are still present.

There is one special “filter” which only penalizes the occurrence of the same molecule and is not part of any of the scaffold filters above. This penalty is recommended for the Mol2Mol generator. The user can adjust the penalizing factor to be between 0 and 1.

#### Inception

Inception, also known as experience replay, can have a profound impact on the learning rate and sampling of desired molecules [7, 59] (see also Additional file for a demonstration). In REINVENT it is a mechanism to memorize the highest scoring molecules and use those scores to contribute to the loss in addition to the loss computed from the scores of the currently sampled batch. This means that the total loss is calculated from two parts: batch loss and inception loss. The number of molecules contributing to the inception loss can be adjusted by the user as well as the number of randomly sampled molecules from the memory to be used in computing the inception loss. Currently, this memory is only available for the original Reinvent molecular generator (see below).

The inception memory can be seeded with SMILES strings provided by the user to guide the RL into a desired part of the chemical space. It should be noted, that if these molecules do not score highly with the currently chosen scoring function, the molecules will be removed from the memory possibly very early on in the run. As the RL run progresses and generates better scoring molecules in each successive step this is generally to be expected. This also means that, depending on the size of the inception memory and the number of sampled SMILES strings from the memory, the total loss and thus eventually the generation of new molecules starts to be dominated by the highest scoring compounds in the memory. The replay memory will either not at all or only marginally be updated in longer RL runs.

#### Other parameters

The user can adjust the batch size which is the number of SMILES strings sampled in each step. While this parameter can be changed to influence the learning rate in an RL run it should be noted that the batch size will also influence the convergence of the stochastic gradient algorithm (Adam) [60, 61].

Randomizing SMILES can be switched on benefiting LibInvent and LinkInvent runs where the priors were trained with randomized SMILES to improve generalizability of the sampled chemical space and prevent overfitting [51]. Randomizing SMILES is a form of data augmentation which can help to build robust models with smaller data sets [62].

### Run modes

**Fig. 3:**
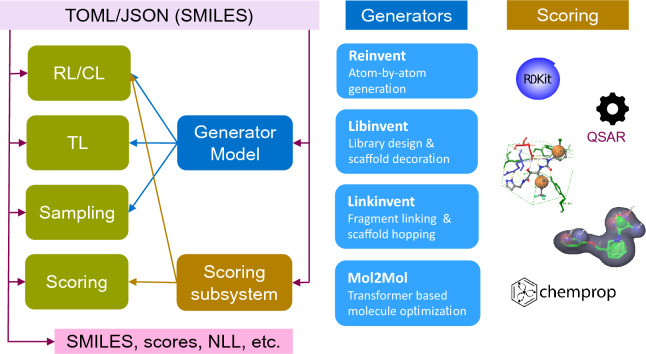
Information flow in REINVENT 4 for all run modes (green boxes) depicted in the left row. Also shown are the supported generators and the scoring subsystem. A input configuration file in TOML or JSON format controls all aspects of the software. The configuration file may contain “seed” SMILES for the Lib/Linkinvent and Mol2Mol2 generators. Input SMILES strings are needed for staged learning, TL and scoring. NLL is the negative log–likelihood as defined in Eqs. 3 and 4

REINVENT 4 supports various “run modes” which are briefly described here. All run modes can either run on a GPU or a CPU. TensorBoard output is written for transfer and reinforcement learning, respectively. Figure 3 summarizes the basic flow of information in REINVENT 4. Input configuration file examples in the TOML format are listed in the additional file material. SMILES are canonicalized with RDKit and normalized [20, 46, 47, 54] before passed on to the learning algorithms. Only the Mol2Mol prior (see "Priors") supports stereochemistry.

#### Scoring

This run mode passes input SMILES strings to the scoring subsystem (see "Scoring Subsystem") and returns the results in a CSV file. The CSV file contains columns for the SMILES strings, the total score and each individual component score both in “raw” (unmodified i.e. not transformed) and transformed form. Duplicate input SMILES strings will not be removed thus the CSV file may contain identical rows. An example of an input file can be found in the (Additional file 1) Listing S6.

#### Sampling

This run mode generates molecules given a model pruduced by either TL or RL. No input SMILES are needed for Reinvent, a scaffold is needed for Libinvent, two warheads for Linkinvent and an input molecule for Mol2Mol. The output is a CSV file containing the sampled SMILES, the input or fragment SMILES (where applicable) and the *negative* log likelihood (which is a *positive* mangitude) for the sampled SMILES. Output SMILES will be canonicalized and duplicates can be removed. Mol2Mol supports either multinomial sampling (with temperature) or beam search.

#### Transfer learning (TL)

TL optimizes a more general model to generate molecules that are closer to a defined set of input molecules. The user provides a prior and a SMILES file e.g. a chemical series. TL will compute the negative log likelihood from the molecules and computes the loss from the resulting mean negative log likelihood over all molecules. This will drive the current prior towards a model which is increasingly closer to the provided molecules.

As this is prone to overfitting (the model will start to generate molecules identical to the input SMILES strings) a validation set of SMILES can be provided which enables the user to monitor the validation loss. Training/validation set split is currently the responsibility of the user. The output is a new model file which can be used for RL or sampling.

The user can set the desired number of epochs, how often the current state of the model should be written out and the batch size. Mol2Mol allows in addition to set the similarity type (see Table 4) and its upper and lower threshold.

#### Staged learning

This is basically curriculum learning [58] (CL) which in REINVENT 4 is implemented as a multi–stage RL. The main purpose is to allow the user to optimize a prior model conditioned on a calculated target profile by varying the scoring function in stages. Typically this would be used to gradually “phase–in” computationally more expensive scoring functions e.g. before docking is enabled it may make sense to first filter the molecules with custom alerts and scoring functions that assess the drug–likeness of the generated molecules. Custom alerts are a set of SMARTS patterns of unwanted chemistries.

Multiple stages can be provided at once (automatic CL). After each stage a checkpoint file is written to disk which can be used for the next stage (manual CL). A stage terminates if the supplied maximum score or the maximum number of steps is reached. In the latter case all stages will be terminated.

Staged learning requires both a prior and an agent model. The prior is only being used as a reference, see discussion in "Generating Molecules". The agent is the model that is being trained in the run. At the beginning of a staged learning run prior and agent will typically be the same model file. When a run terminates, either because the termination criterion has been reached or the user terminates the run explicitly (Ctrl–C) a checkpoint file representing the current state of the agent will be written to disk. This checkpoint can be reused as the agent later.

Just as for sampling the user needs to supply a file with a molecule or a fragment SMILES string depending on the desired generator. This is not needed for Reinvent which constructs molecules from scratch. Mol2mol allows both beam search and multinomial sampling strategies.

The user can set the batch size and whether input SMILES should be randomized or generated sequences should be unique (this form of de–duplication is a feature from previous versions of REINVENT and is kept for backward compatibility). The available learning strategies (explained in "RL Learning Strategy") can be tuned with $\sigma$ to control the contribution of the total scoring function to the augmented log-likelihood, see Eq. 6, and the learning rate. Diversity filter and inception are both optional.

All scaffold diversity filters need a parameter for the size of each scaffold bucket. Each molecular SMILES string is stored in a single memory. Both memories are subject to a minimum score parameter that is only if the total score exceeds this value scaffolds and molecules are stored. A minimum Dice similarity is needed for the similar scaffold filter. A penalty multiplier is used for penalizing the total score of a SMILES string in the penalize same SMILES string filter.

Inception may be seeded optionally with a list of SMILES strings, the size of the memory and how many random samples should be included in each step can be adjusted.

For each stage a scoring profile can be defined which can also be read in from a separate file for easier reuse. The supported formats are TOML and JSON. The stage is terminated either when a maximum score threshold is exceeded or the maximum number of steps is reached. In the former case the run proceeds to the next stage (if present). In the latter case the whole run is terminated. The rationale is that the user then inspects if the run should proceed or not because not reaching the score threshold may be a sign that there is a problem with that stage. The user can also enforce a minimum number of steps before the termination criterion is checked.

The results are written into a CSV file similar to the scoring run mode but one file is created for each stage. The user can define a prefix for the CSV file name that is then appended with a number for the current stage. The CSV file contains columns for the negative log likelihoods for prior, agent and the augmented likelihood (Eq. 6). Further columns are for the generated SMILES string, the total and individual component scores (both raw and transformed) and a final column records the current step number (epoch).

### Molecule generators

**Fig. 4:**
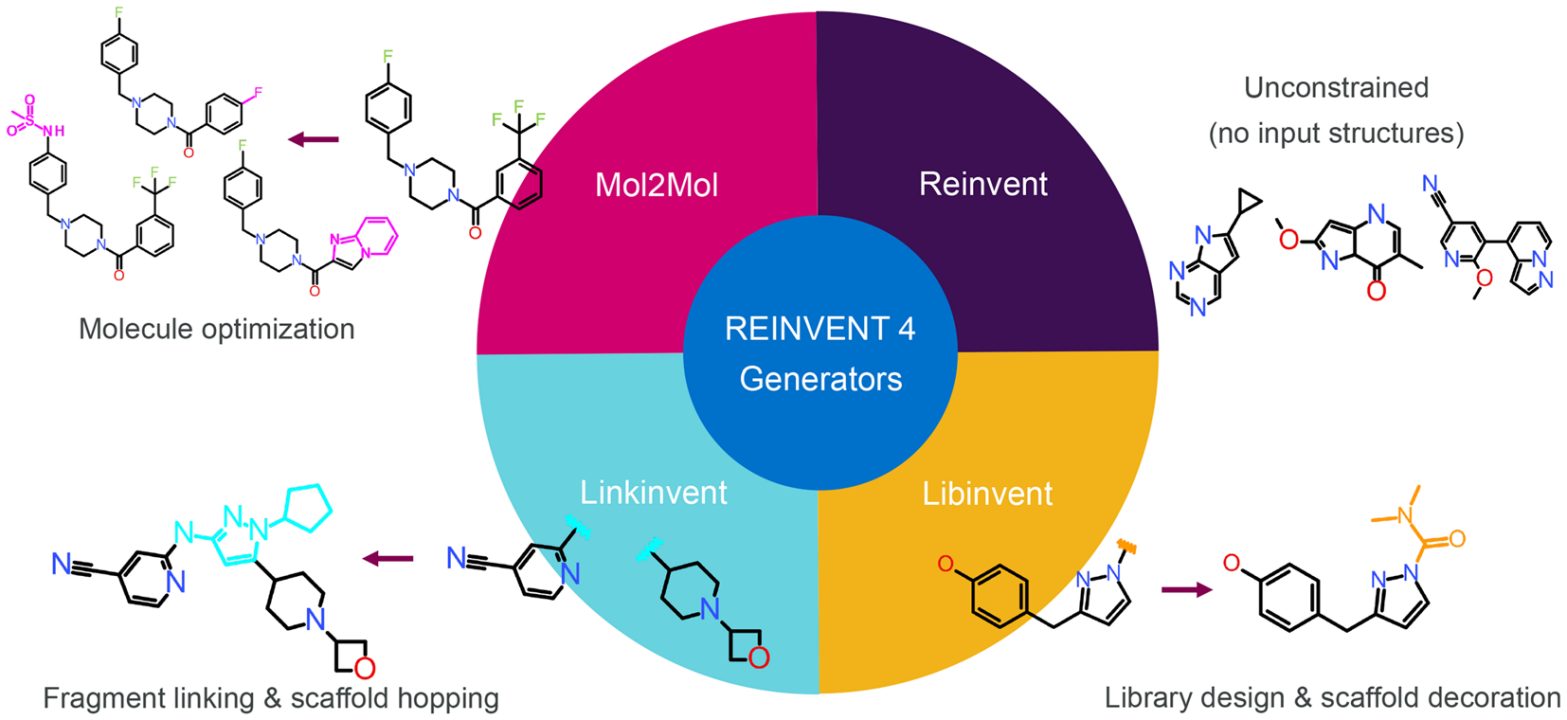
The four types of molecular generators in REINVENT 4 illustrating how they work. Reinvent creates new molecules *de novo* i.e. from scratch, Libinvent decorates a scaffold, Linkinvent identifies a linker between two fragments and Mol2Mol optimizes molecules within a user defined similarity

REINVENT 4 supports several molecule generators which will be briefly describe here, see Fig. 4. A generator is a fundamental algorithm which creates new molecules considering certain constraints. The project name of the generator as described in previous publications will be given in parentheses.

1. De novo design [19, 20]. This unconstrained and unrestrained generator builds molecules in sequence atom–by–atom using an RNN. This is the classical *de novo* algorithm described in the very first publication of REINVENT [19]. (Reinvent)
2. R–group replacement and library design [46]. A scaffold is supplied to the RNN based generator serving as a template and constraint in building the new molecule. The generator will decorate this scaffold with suitable R–groups. Up to four attachment points are supported. Naturally this generator can also be used to create AI guided libraries. (Libinvent)
3. Fragment linking and scaffold hopping [47]. Two “warheads” are supplied to the RNN based generator as constraints. The generator will create a suitable linker joining the two warheads. Generally, the linker can be any type of scaffold (subject to the training set of the prior). (Linkinvent)
4. Molecular optimization [22, 52]. A molecule is supplied to the generator as *restraint*. The generator will find a second molecule within a defined similarity. Depending on the similarity radius the molecule will be relatively similar to the supplied molecule but, importantly, the scaffold can change within the limits of the given similarity. (Mol2Mol)

### Scoring subsystem

Reinforcement learning is an optimization algorithm in machine learning which rewards a desired behaviour. In this context it means that a molecule is optimized with respect to a user defined aggregation of scoring functions which is fed into Eq. 6. REINVENT 4 supports an extensive array of scoring functions as summarized in Table 2. Most scoring functions have multiple, so–called “endpoints”. This can be used for instance to provide several SMARTS patterns e.g. to GroupCount or to compute both inertial moment ratios in the PMI function. Multi–task models are another natural fit for this mechanism allowing the choice of a desired subset.

Scores for each SMILES string will be cached on a per–component basis to avoid the re–computation of scores. All SMILES strings are passed to the subsystem in RDKit canonical form as the priors do not necessarily generate SMILES in canonical form. Duplicates are marked as such and handled by the caching mechanism. Chiral information (when using Mol2Mol) is retained. Each scoring component may transform the SMILES into the form that is needed for the underlining model. This is important as the model may have been trained or operates on a different canonicalization scheme.

It is worth noting that custom alerts and reaction filter act as a global filter and are not components. What this means is that, effectively, the total score will be multiplied with the outcome of the filter, either 1 for passed or 0 for not passed. For efficiency reasons this also implies that SMILES that do not pass those filters will not be subjected to score evaluation and consequently *all* component scores will be zero. Furthermore, there is currently one penalty component: matching substructure which globally applies the penalty factor to the final total score.

Many of the scoring functions cover various physicochemical properties from the RDKit toolkit including Lipinski’s rule–of–five [63] and QED [64]. Special fragment versions of these are available for Linkinvent so to be able to separately score the linker in addition to length scores. Docking is handled with the generic interface DockStream [65] that supports AutoDock Vina [66, 67], rDock [68], Hybrid [69], Glide [70] and GOLD [71]. Quantitative Structure-Activity Relationship (QSAR) models are handled with Qptuna (to be published). ChemProp [72, 73] provides an alternative using directed message-passing neural networks (D-MPNN) for model building. General workflows can still be created with ICOLOS [74] but it will be superseded with the newer workflow manager Maize (to be published). There are also generic interfaces for a REST service calling external processes which allows programming entirely arbitrary scoring components. Shape similarity can be computed with ROCS [75]. Estimation of synthesizability can be carried out with the SA score [76]. Matched molecular pairs can be used via mmpdb [77].

Each scoring function result can be arbitrarily modified with a transformation function to compress scores to between 0 and 1. A list of transforms is given in Table 3. A weight needs to be set for each endpoint to determine its relative importance with respect to the other components.

All components of a scoring function are finally aggregated into a single total score (*a priori* scalar objective [78, 79]). At the moment aggregation is done either via a weighted arithmetic mean or a weighted geometric mean.

The scoring subsystem implements a simple plug–in mechanism (as Python namespace packages) which allows easy addition of scoring components. Basically, new code only needs to be dropped into an existing plugin directory following a code template, see SI for details. None of the original REINVENT 4 code would need to be changed.

**Table 2:** Summary of REINVENT 4 scoring functions

Component name^1^	Description
Qed	QED drug-likeness score (RDKit)
SlogP	Crippen SLogP (RDKit)
MolecularWeight	Molecular weight (RDKit)
TPSA	Topological polar surface area (RDKit)
GraphLength	Topological distance (RDKit)
NumAtomStereoCenters	Number of stereo centers (RDKit)
HBondAcceptors	Number of hydrogen bond acceptors (RDKit)
HBondDonors	Number of hydrogen bond donors (RDKit)
NumRotBond	Number of rotatable bonds (RDKit)
Csp3	Fraction of sp3 carbons (RDKit)
Numsp	Number of sp hybridized atoms (RDKit)
Numsp^b^	Number of sp2 hybridized atoms (RDKit)
Numsp^c^	Number of sp3 hybridized atoms (RDKit)
NumHeavyAtoms	Number of heavy atoms (RDKit)
NumHeteroAtoms	Number of hetero atoms (RDKit)
NumRings	Number of total rings (RDKit)
NumAromaticRings	Number of aromatic rings (RDKit)
NumAliphaticRings	Number of aliphatic rings (RDKit)
GroupCount ^d^	Count how many times the SMARTS pattern is found (RDKit)
PMI ^d^	Principal moment of inertia to assess dimensionality (RDKit)
TanimotoDistance	Tanimoto distance using the Morgan fingerprint (RDKit)
MatchingSubstructure	*penalty* applied to final score when SMARTS pattern is found (RDKit)
ReactionFilter ^d^	Reaction *filter* for Libinvent, applied to total score (RDKit)
CustomAlerts	SMARTS substructure *filter* applied to the total score (RDKit)
DockStream	Docking interface [65] (see text for supported docking software)
Lcolos	Generic interface to Icolos workflow manager [74]
Maize ^d^	Generic interface to Maize workflow manager^1^ (replaces Icolos)
Qptuna ^d^	QSAR models with Qptuna^3^
ChemProp ^d^	ChemProp D–MPNN models [72, 73]
MMP ^d^	Matched molecular pairs [77]
ROCSSimilarity	ROCS [75]
SAScore ^d^	Synthesizability score [76]
ExternalProcess ^d^	Generic component to run an external process for scoring
REST	Generic REST interface

^a^The name of the scoring component in the TOML/JSON configuration file.

^b^https://github.com/MolecularAI/maize

^c^To be published

^d^New in REINVENT 4

**Table 3:** Summary of REINVENT 4 transforms

Transform	Description
Sigmoid	S–shaped logistic function
Reverse_sigmoid	Reverse sigmoid function
Double_sigmoid	Two–sided sigmoid function
Right_step	Heaviside step function, can be shifted along x
Left_step	Left–sided step function
Step	Two–sided step function
Value_mapping	Maps a categorical value (string) to a user–supplied number

### Priors

REINVENT 4 provides a range of off-the-shelf ready–made priors. These are pre–trained on ChEMBL [80] (except of the Mol2Mol prior which is trained on PubChem) and specific to each generator. Table 4 summarizes all currently available priors. Listing 10 (Additional file 1) lists all recognized tokens of the priors. All priors support the same atoms (elements). The main differences between the priors are ring sizes and that Mol2Mol supports and generates chiral centers at C and (quarternary) N and double bond isomers.

**Table 4:** Summary of REINVENT 4 priors. Mol2Mol comes with six different priors with pairs trained on different types of similarity

Generator	Dataset	Notes
Reinvent	ChEMBL 25	Published in Ref. [19, 20]
Libinvent	ChEMBL 27	Published in Ref. [46]
Linkinvent	ChEMBL 27	Published in Ref. [47]
Mol2Mol	ChEMBL 28	Published in Ref. [22]
		Similarity^a^
		Medium similarity^b^
		High similarity^c^
		Scaffold^d^
		Generic scaffold^e^
		Matched molecular pairs^f^
Mol2Mol	Pubchem^g^	Published in Ref. [54]
		Similarity^g^

^a^Tanimoto similarity $$\ge$$ 0.5.

^b^.5 $$\le$$ Tanimoto similarity < 0.7.

^c^Tanimoto similarity $$\ge$$ 0.7.

^d^Molecules sharing the same Murcko scaffold (RDKit).

^e^Molecules sharing the same unlabelled Murcko scaffold.

^f^Matched molecular pairs have been extracted with mmpdb [77].

^g^Pubchem was collected in December 2021.

^h^Tanimoto similarity $\ge$ 0.5 on ECFP4 fingerprints with counts

### Software

The software is available from https://github.com/MolecularAI/REINVENT4 and released under the permissive Apache 2.0 license. REINVENT 4 is being developed with Python 3. The currently required minimum version is 3.10. We use the machine learning framework Pytorch in version 1.x but initial tests have shown that the newer version 2.0 works as well. For chemoinformatic manipulations we use RDKit in version 2022.9. In fact, any recent version of RDKit should be sufficient. For visualisation REINVENT supports TensorBoard [81] which logs generated molecules and various statistics from RL and sampling runs as easy to interpret graphs. REINVENT 4 is not principally backward compatible with previous versions because the layout of the input configuration has changed. It is still possible to use JSON as input file format but version 4 now also supports TOML (https://toml.io/) which tends to be more user friendly. The configuration file controls almost all aspects of REINVENT 4 (see SI for example inputs).

Just like in previous versions REINVENT 4 is a command line tool (see "Installation"). A few command line options are available (see +–help+ for details), most notably are the ones for writing logging information to a file (+stderr+ by default) and the choice of format for the input configuration file (TOML by default or JSON). The logging information shows timestamped information about software versions used, parameter settings and setup as well as some basic statistics of the run including memory usage. The output will depend on the particular run mode chosen, see "Run Modes". The random seed can be set for PyTorch and Numpy (efficient vector and matrix handling) to aid in reproducibility Additional files: 1, 2

### Installation

Detailed installation instructions are provided in the repository in the file +README.md+. In short, the user needs to create a basic conda environment. This environment is then populated with REINVENT 4 and dependent packages using +pip+. Versioning of dependencies is controlled through a lock file to guarantee a functioning environment out–of–the–box. The installation will create an entry point into the main script of REINVENT and generate a simple wrapper that can be called on the command line as +reinvent+.

### Documentation

The new TOML format is described in several markdown documents located in +configs/toml+. Details are there given on the various option for each run mode and generator settings. The Supplement provides annotated listings which can also be found in the directory.

## Case study

We provide a simple example to demonstrate some of the key functionalities in REINVENT 4 (see Additional file 2 for input data). To this end we describe a hypothetical virtual screening exercise to find novel Phosphoinositide-dependent kinase-1 (PDK1) inhibitors. A more detailed study has been published previously [58] which itself is based on the original structure–based design work of Angiolini et al. [82]. In contrast to our previous experiment, we consider a simple structure–based design setting where we seek to identify putative PDK1 binders. We define a simple target profile consisting of a docking component and the QED score [64] to approximate drug–like properties. The generated compounds where docked without constraints to PDB crystal structure 2XCH using DockStream [65] with Ligprep and Glide [70]. Here, we arbitrarily consider any molecule generated with a docking score $\le -8$ kcal/mol and QED $\ge 0.7$ as a favourable compound.

Starting from the standard Reinvent prior, we run 50 epochs of staged learning with a batch size of 128 and the two scoring components. Input configurations are provided in the SI (Additional file 1: Listings S8 and S9) and the required grid and files for docking are available in the electronic SI.

**Fig. 5:**
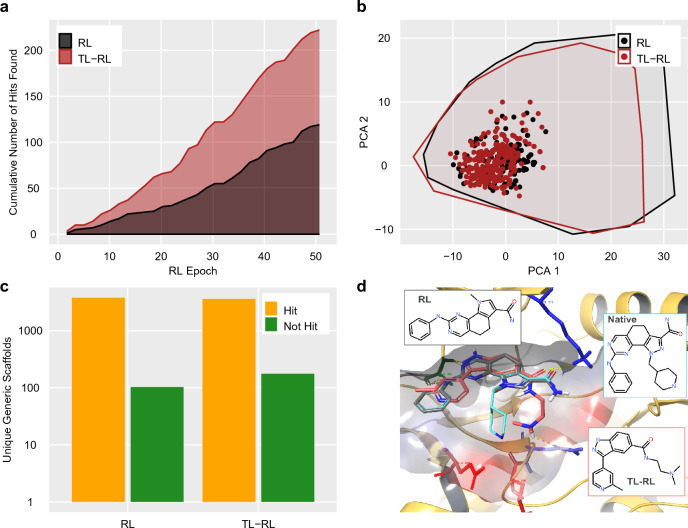
Demonstration of a simple structure–based drug design in REINVENT 4 using a crystal structure for PDK1 (PDB ID 2XCH). The cumulative number of hits identified over 50 epochs are shown **a** for reinforcement learning starting from the prior (RL, black) or from a transfer learning agent (TL-RL, red). The diversity of the hits generated is compared using principal component analysis (PCA) based on 2D RDKit descriptors**b** and by counting the number of distinct hit and not–hit generic scaffolds **c**. For the PCA plot, we show hits as colored circles and include the convex hulls of all generated compounds as polygons **b**. **d** The predicted binding pose in the PDK1 binding site (based on PDB 2XCH) for the best scoring idea from each method are shown with a stick representation, contrasted with the native ligand in cyan. The docking scores for the poses are as follows: $-10.1$ kcal/mol (RL) and $-10.1$ kcal/mol (TL-RL). The protein is represented as a cartoon with key binding site residues (ALA 162/green, LYS 111/blue, GLU166/red, GLU209/red, ASN 210/blue) shown in a stick representation, with a transparent binding site surface overlaid. 2D inserts show the structure of the ligands. Hits are defined as molecules with a docking score $\le -8$ kcal/mol and QED $\ge 0.7$

Despite the rather short RL run, we are able to generate 119 hits from 6400 ($128 \times 50$) total generated molecules for a hit rate of 1.9 % (Fig. 5a). However, the productivity of RL agents increases with epoch (see for example Ref [57]), being 2.8% in the last 20 epochs. These hits are spread across 103 generic Bemis–Murcko scaffolds [83], indicating high diversity (Fig. 5b and c). Remarkably, the top scoring hit is a pyrroloquinazoline that is extremely similar to the native pyrazoloquinazoline core. This generated molecule is predicted to adopt an identical binding pose, including the hinge interaction ALA 162 and an amide that interacts with LYS 111, seen in the native structure (Fig. 5d).

In order to demonstrate the potential advantages of TL, we obtained a list of 315 congeneric pyridinon–bearing compounds shown to be active against PDK1 as per PubChem Assay AID1798002. We selected this set because it is the largest (in terms of number of compounds tested in a single assay) reported in PubChem against PDK1. A more careful study could consider multiple assays or more intentionally curated relevant chemistry.

After running 10 epochs of TL we repeated the structure–based design exercise starting with this agent. Although the compounds in the series are not closely related to the native pyrazoloquinazoline inhibitor the TL agent is nearly twice as productive as the baseline RL agent over 50 epochs, finding 222 hits with a 3.5% hit rate (Fig. 5a). The diversity is high (Fig. 5b, c) with 176 unique generic scaffolds identified. The pose corresponding to the best docking score contains an imidazole core that makes the same interaction with LYS 111 as the native ligand, and also positions a basic nitrogen close to ASN 210 and GLU 208 which makes it a plausible design hypothesis. However, we observe the hinge interaction with ALA 162 is not complete in this case due to a missing donor (Fig. 5d). This could be addressed through the addition of constraints in the docking grid as was done in [58].

## Conclusion

The technical details and basic usage of the new version 4 of the AI molecular design software REINVENT have been described. The tool is both a continuation of previous releases and a major update in functionality including staged learning, transformer models, consistent framework of optimization algorithms and a reworked scoring subsystem fit for future challenges. We hope that the AI in chemistry community will greatly benefit from the release of a reference implementation of a molecular generation software including releasing the software as open–source and making all documentation available to guide the user. We hope that the release will contribute to increased transparency around AI–driven molecular design and the released software be used as a reference implementation for educational purposes as well as spur further innovation in generative AI for molecular design. The software is available from https://github.com/MolecularAI/REINVENT4.

### Supplementary Information

**Additional file 1: **Additional validation results, input file examples, supported tokens.

**Additional file 2: **ZIP archive containing data used in the case study: docking grid, DockStream configuration, SMILES used for transfer learning.

## Data Availability

The source code is available from https://github.com/MolecularAI/REINVENT4.
