# Supplementary material for: Reinvent 4: Modern AI–driven generative molecule design
Source: J Cheminform. 2024 Feb 21;16:20. doi: 10.1186/s13321-024-00812-5 (PMC10882833; doi:10.1186/s13321-024-00812-5)
Supplement: Supplementary file 1 — Additional file 1: Additional validation results, input file examples, supported tokens. [file 13321_2024_812_MOESM1_ESM.pdf]

REINVENT 4: Modern Generative AI for Molecular Design (Supplement)

Hannes H. Loeffler[1*], Jiazhen He[1], Alessandro Tibo[1], Jon Paul Janet[1], Alexey Voronov[1], Lewis Mervin[2], Ola Engkvist[1]

[1*]Molecular AI, Discovery Sciences, R&D, AstraZeneca, Gothenburg, Sweden.

[2]Molecular AI, Discovery Sciences, R&D, AstraZeneca, Cambridge, UK.

*Corresponding author(s). E-mail(s): hannes.loffler@astrazeneca.com;

1 Effect of Inception and Scaffold Diversity Filter

Figure S1 summarizes how inception and scaffold diversity filter (DF) affect Reinforcement Learning (RL). All results are taken as the average over 5 independent runs. The highlighted areas denote the standard deviation for each data point.

The data (orange and blue lines)in S1a show how inception leads to finding significantly more compounds with a smaller binding free energy. When inception is switched on, DF displays higher ΔG values. With DF on RL is forced to create more diverse structures and does not allow the run to exploit a smaller number of scaffolds.

Subfigure S1b displays the fraction of valid SMILES found in each RL step. When DF is on the number of valid SMILES drops relative to when DF is off.

In S1c we see the effect of inception on the number of unique scaffolds found (DF is on). Inception leads to a larger diversity in sampled structures.

Subfigure S1d shows how inception affects how many unique scaffolds are found more than N times. N is the size of the DF memory and was taken as 10 in this case. Clearly, with inception RL samples larger numbers of scaffolds in line with S1c.

The scoring function used for this examples is composed of QED, custom alerts, number of stereocentres (set to zero) and binding free energy. The latter is built with a QSAR–like response model derived from about 10 000 MM–PBSA calculations of ligands bound to the main protease of SARS–2. The data here only serves as a demonstration. Detailed results will be published elsewhere. The batch size was set to 100, the RL learning strategy was DAP with $\sigma = 128$ and a learning rate of 0.0001, DF was of type Murcko scaffold with a memory size of 10 and minimum score threshold

for storage of 0.4 and the inception memory size was set to 50 with a sample size of 10. RL was executed for 300 steps.

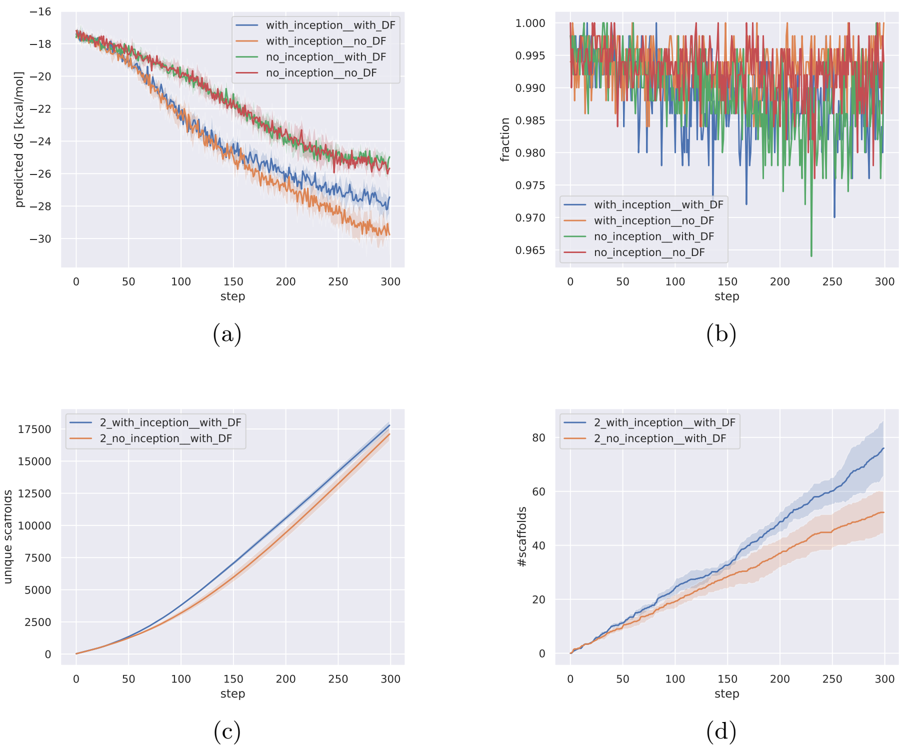

Fig. S1: Effect of inception and scaffold diversity filter. a) Average binding free energy, b) Fraction of valid SMILES found per batch (typically above 95%), c) Number of unique scaffolds, d) Number of scaffolds found more than 10 times.

2 Input configuration examples

Listing S1: Staged learning input example.

```
# REINVENT4 TOML input example for reinforcement/curriculum learning
#
#
# Curriculum learning in REINVENT4 is a multi-stage reinforcement
    learning
# run. One or more stages (auto CL) can be defined. But it is also
```

```
# possible to continue a run from any checkpoint file that is generated
# during the run (manual CL). Currently checkpoints are written at the end
# of a run also when the run is forcefully terminated with Ctrl-C.


run_type = "staged_learning"
use_cuda = true # run on the GPU if true, on the CPU if false
tb_logdir = "tb_logs" # name of the TensorBoard logging directory
json_out_config = "_staged_learning.json" # write this TOML to JSON

[parameters]

# Uncomment one of the comment blocks below. Each generator needs a model
# file and possibly a SMILES file with seed structures. If the run is to
# be continued after termination, the agent_file would have to be replaced
# with the checkpoint file.

use_checkpoint = false # if true read diversity filter from agent_file
summary_csv_prefix = "staged_learning" # prefix for the CSV file

## Reinvent
prior_file = "models/reinvent.prior"
agent_file = "models/reinvent.prior"

## LibInvent
#prior_file = "models/libinvent.prior"
#agent_file = "models/libinvent.prior"
#smiles_file = "scaffolds.smi" # 1 scaffold per line with attachment points

## LinkInvent
#prior_file = "models/linkinvent.prior"
#agent_file = "models/linkinvent.prior"
#smiles_file = "warheads.smi" # 2 warheads per line separated with '|'

## Mol2Mol
#prior_file = "models/mol2mol_scaffold_generic.prior"
#agent_file = "models/mol2mol_scaffold_generic.prior"
#smiles_file = "mol2mol.smi" # 1 compound per line
```

```
#sample_strategy = "multinomial" # multinomial or beamsearch (deterministic)
#distance_threshold = 100

batch_size = 64       # network

unique_sequences = true # if true remove all duplicates raw sequences in each step
                       # only here for backward compatibility
randomize_smiles = true # if true shuffle atoms in SMILES randomly


[learning_strategy]

type = "dap"    # dap: only one supported
sigma = 128     # sigma of the RL reward function
rate = 0.0001   # for torch.optim


[diversity_filter] # optional, comment section out or remove if unneeded
                  # NOTE: also memorizes all seen SMILES

type = "IdenticalMurckoScaffold" # IdenticalTopologicalScaffold,
                              # ScaffoldSimilarity, PenalizeSameSmiles
bucket_size = 25              # memory size in number of compounds
minscore = 0.4                # only memorize if this threshold is exceeded
minsimilarity = 0.4           # minimum similarity for ScaffoldSimilarity
penalty_multiplier = 0.5      # penalty factor for PenalizeSameSmiles


# Reinvent only: guide RL in the initial phase
#[inception] # optional, comment sectionout or remove if unneeded

#smiles_file = "sampled.smi" # "good" SMILES for guidance
#memory_size = 100 # number of total SMILES held in memory
#sample_size = 10 # number of SMILES randomly chosen each epoch


### Stage 1
### Note that stages must always be a list i.e. double brackets
[[stage]]

```

```
chkpt_file = 'test1.chkpt' # name of the checkpoint file, can be reused as agent

termination = "simple" # termination criterion fot this stage
max_score = 0.6 # terminate if this total score is exceeded
min_steps = 25 # run for at least this number of steps
max_steps = 100 # terminate entire run when exceeded

[stage.scoring]
type = "geometric_mean" # aggregation function

[[stage.scoring.component]]
# Custom alerts if used in a custom_product filter out unwanted groups
[stage.scoring.component.custom_alerts]

[[stage.scoring.component.custom_alerts.endpoint]]
name = "Unwanted SMARTS" # user chosen name for output
weight = 0.79 # weight to fine-tune the relevance of this component

# parameters for the component:
# a list of unwanted SMARTS(!) to be scored as zero
params.smarts = [
    "[*;r8]",
    "[*;r9]",
    "[*;r10]",
    "[*;r11]",
    "[*;r12]",
    "[*;r13]",
    "[*;r14]",
    "[*;r15]",
    "[*;r16]",
    "[*;r17]",
    "[#8][#8]",
    "[#6;+]",
    "[#16][#16]",
    "[#7;!n][S;!$(S(=O)=O)]",
    "[#7;!n][#7;!n]",
    "C#C",
    "C(=[O,S])[O,S]",
    "[#7;!n][C;!$(C(=[O,N])[N,O])][#16;!s]",
    "[#7;!n][C;!$(C(=[O,N])[N,O])][#7;!n]",
    "[#7;!n][C;!$(C(=[O,N])[N,O])][#8;!o]",
    "[#8;!o][C;!$(C(=[O,N])[N,O])][#16;!s]",
    "[#8;!o][C;!$(C(=[O,N])[N,O])][#8;!o]",
    "[#16;!s][C;!$(C(=[O,N])[N,O])][#16;!s]"
```

```
]

[[stage.scoring.component]]
[stage.scoring.component.MolecularWeight]

[[stage.scoring.component.MolecularWeight.endpoint]]
name = "Molecular weight" # user chosen name for output
weight = 0.342 # weight to fine-tune the relevance of this component

# A transform ensures that the output from the scoring component ranges
# from 0 to 1 to serve as a proper score. Here we use a double sigmoid
# to transform weights into the range 200-500 a.u.
transform.type = "double_sigmoid"
transform.high = 500.0
transform.low = 200.0
transform.coef_div = 500.0
transform.coef_si = 20.0
transform.coef_se = 20.0


### Stage 2
# Alternatively only the first stage above can be run and the new input file
# sets agent_file = 'test1.chkpt'.

[[stage]]

chkpt_file = 'test2.chkpt'

termination = "simple"
max_score = 0.7
min_steps = 10
max_steps = 100

[stage.scoring] # the scoring components can be read from a score file
type = "geometric_mean" # aggregation function
filename = "stage2_scoring.toml" # file with scoring setup for this stage
filetype = "toml" # file format: TOML or JSON, no default, must be present


### Stage 3
# just as above
```

Listing S2: Scoring components read from a separate file.

```
### Stage 2 scoring components
[[component]]
[component.QED]

[[component.QED.endpoint]]
weight = 0.5 # user chosen name for output
name = "QED Score" # weight to fine-tune the relevance of this component
```

Listing S3: Example scaffold file for REINVENT 4 LibInvent.

```
# One scaffold per line
# Each scaffold must be annotated by 1 to 4 '*'s to locate the
#   attachment points followed with the label number after ':'

[*:1]Cc2ccc1cncc(C[*:2])c1c2
[*:1]Cc2cnc1cncc(C[*:2])c1c2
```

Listing S4: Example warhead file for REINVENT 4 LinkInvent.

```
# One warhead pair per line
# Each warhead must be annotated with '*' to locate the attachment points
# The two warheads must be separated by the pipe symbol '|'

Oc1cncc(*)c1|*c1ccoc1
Nc1c([F])ccc(*)c1|*c1c([Cl])cnc1
```

Listing S5: Sampling input example.

```
# REINVENT4 TOML input example for sampling
#


run_type = "sampling"
use_cuda = true # run on the GPU if true, on the CPU if false
json_out_config = "_sampling.json" # write this TOML to JSON


[parameters]

# Uncomment one of the comment blocks below. Each generator needs a model
```

```
# file and possibly a SMILES file with seed structures.

## Reinvent: de novo sampling
model_file = "models/reinvent.prior"

## LibInvent: find R-groups for the given scaffolds
#model_file = "models/libinvent.prior"
#smiles_file = "scaffolds.smi" # 1 scaffold per line with attachment points

## LinkInvent: find a linker/scaffold to link two fragments
#model_file = "models/linkinvent.prior"
#smiles_file = "warheads.smi" # 2 warheads per line separated with '|'

## Mol2Mol: find molecules similar to the provided molecules
#model_file = "models/mol2mol_medium_similarity.prior"
#smiles_file = "mol2mol.smi" # 1 compound per line
#sample_strategy = "beamsearch" # multinomial or beamsearch (deterministic)
#temperature = 1.0 # temperature in multinomial sampling
#tb_logdir = "tb_logs" # name of the TensorBoard logging directory

output_file = 'sampling.csv' # sampled SMILES and NLL in CSV format

num_smiles = 157 # number of SMILES to be sampled, 1 per input SMILES
unique_molecules = true # if true remove all duplicatesd canonicalize smiles
randomize_smiles = true # if true shuffle atoms in SMILES randomly
```

Listing S6: Scoring input example.

```
# REINVENT4 TOML input example for scoring
#

run_type = "scoring"
output_csv = "scoring.csv" # run on the GPU if true, on the CPU if false
json_out_config = "_scoring.json" # write this TOML to JSON

[parameters]

smiles_file = "compounds.smi" # SMILES file with 1 molecule per line


[scoring]
```

```
type = "geometric_mean" # or arithmetic_mean
parallel = false # do not run scoring components in parallel

[[scoring.component]]
# Custom alerts if a filter of unwanted groups
[scoring.component.custom_alerts]

[[scoring.component.custom_alerts.endpoint]]
name = "Alerts" # user chosen name for output
# no weight because as a filter it will be applied globally

params.smarts = [
    "[*;r8]",
    "[*;r9]",
    "[*;r10]",
    "[*;r11]",
    "[*;r12]",
    "[*;r13]",
    "[*;r14]",
    "[*;r15]",
    "[*;r16]",
    "[*;r17]",
    "[#8][#8]",
    "[#6;+]",
    "[#16][#16]",
    "[#7;!n][S;!$(S(=O)=O)]",
    "[#7;!n][#7;!n]",
    "C#C",
    "C(=[O,S])[O,S]",
    "[#7;!n][C;!$(C(=[O,N])[N,O])][#16;!s]",
    "[#7;!n][C;!$(C(=[O,N])[N,O])][#7;!n]",
    "[#7;!n][C;!$(C(=[O,N])[N,O])][#8;!o]",
    "[#8;!o][C;!$(C(=[O,N])[N,O])][#16;!s]",
    "[#8;!o][C;!$(C(=[O,N])[N,O])][#8;!o]",
    "[#16;!s][C;!$(C(=[O,N])[N,O])][#16;!s]"
]

[[scoring.component]]
[scoring.component.QED]

[[scoring.component.QED.endpoint]]
name = "QED"
weight = 0.25 # weight to fine-tune the importance of this component

```

```
[[scoring.component]]
[scoring.component.MolecularWeight]

[[scoring.component.MolecularWeight.endpoint]]
name = "MW"
weight = 0.25

# transforms can be used for any component if desired
transform.type = "double_sigmoid"
transform.high = 500.0
transform.low = 200.0
transform.coef_div = 500.0
transform.coef_si = 20.0
transform.coef_se = 20.0

[[scoring.component]]
[scoring.component.TanimotoDistance]

[[scoring.component.TanimotoDistance.endpoint]]
name = "Tanimoto similarity ECF6"
weight = 0.1

# component specific parameters
params.smiles = [
    "n1(nc(c(c1C)-c2n[nH]c(c2)[C@@]3([C@@H](CN(CC3)Cc4nc5c(c(n4)C)cccc5)O)OC)C)C"
]
params.radius = 3
params.use_counts = true
params.use_features = true


# Multiple end-points for many scoring components: each is a separate
    component
# with its own weight
[[scoring.component]]
[scoring.component.pmi]
name = "PMI 3D-likeness"

[[scoring.component.pmi.endpoint]]
weight = 0.79
params.property = 'npr1' # component specific parameters

[[scoring.component.pmi.endpoint]]
weight = 0.21
params.property = 'npr2' # component specific parameters
```

Listing S7: Transfer Learning input example.

```
# REINVENT4 TOML input example for transfer learning
#
# Focus a given model towards a set of input SMILES. This can also be used to
# start from scratch from and an untrained prior.


run_type = "transfer_learning"
use_cuda = true # run on the GPU if true, on the CPU if false
tb_logdir = "tb_TL" # name of the TensorBoard logging directory
json_out_config = "json_transfer_learning.json" # write this TOML to JSON


[parameters]

num_epochs = 3 # number of steps to run
save_every_n_epochs = 3 # save checkpoint model file very N steps
batch_size = 50
num_refs = 100 # number of reference molecules randomly chosen for similarity
sample_batch_size = 100 # number of sampled molecules chosen for similarity
# Uncomment one of the comment blocks below. Each generator needs a model
# file and possibly a SMILES file with seed structures.

## Reinvent
#input_model_file = "models/reinvent.prior"
#smiles_file = "TL_reinvent_100.smi" # read 1st column
#output_model_file = "TL_reinvent.model"
#validation_smiles_file = "TL_reinvent_100.smi"

## Mol2Mol
input_model_file = "models/mol2mol_scaffold_generic.prior"
smiles_file = "compounds.smi" # read 1st column
output_model_file = "TL_Mol2Mol.model"
validation_smiles_file = "validation_compounds.smi" # read 1st column

# Define the type of similarity and its parameters
pairs.type = "tanimoto"
pairs.upper_threshold = 1.0
pairs.lower_threshold = 0.7
pairs.min_cardinality = 1
```

```
pairs.max_cardinality = 199

### It may not make much sense to use TL for Lib/Linkinvent because
### both methods use constraints which may leave only a small portion
    of
### the molecule to be optimizable
## LibInvent
#input_model_file = "models/libinvent.prior"
#smiles_file = "TL_libinvent_100.smi" # read first 2 columns:
    warheads, linker
#validation_smiles_file = "TL_libinvent_100.smi" # read first 2
    columns: warheads, linker
#output_model_file = "TL_libinvent.model"
#
## LinkInvent
#input_model_file = "models/linkinvent.prior"
#smiles_file = "TL_linkinvent_100.smi" # read first 2 columns:
    scaffold, R-groups
#output_model_file = "TL_linkinvent.model"
#validation_smiles_file = "TL_linkinvent_100.smi" # read first 2
    columns: scaffold, R-groups
```

Listing S8: Structure based drug design example, RL

```
version = 4
run_type = "staged_learning"
use_cuda = true
output_csv = "rl_direct_.csv"
tb_logdir = "tb_rl_direct"

[parameters]
use_checkpoint = false
summary_csv_prefix = "rl_direct"
agent_file = "/models/reinvent.prior"
prior_file = "/models/reinvent.prior"

batch_size = 128
uniquify_smiles = true
randomize_smiles = true

[learning_strategy]

type = "dap"
sigma = 128
rate = 0.0005
```

```

[diversity_filter]

type = "IdenticalMurckoScaffold"
bucket_size = 50
minscore = 0.4

[[stage]]
chkpt_file = 'rl_direct.chkpt'
termination = "simple"
max_steps = 50

[stage.scoring]
type = "geometric_mean"

[[stage.scoring.component]]
[[stage.scoring.component.DockStream.endpoint]]
name = "Docking into 2XCH grid"
weight = 1
params.configuration_path = "dockstream_rl_direct_uncs.json"
params.docker_script_path = "[PATH_TO_DOCKSTREAM]/docker.py"
params.docker_python_path = "[PATH_TO_DOCKSTREAM_ENV]/python"
transform.type = "reverse_sigmoid"
transform.high = -7
transform.low = -13.5
transform.k = 0.2

[[stage.scoring.component]]
[stage.scoring.component.QED]
[[stage.scoring.component.QED.endpoint]]
name = "QED"
weight = 1
```

Listing S9: Structure based drug design example, TL

```
# Transfer learning on a set of related active compounds

run_type = "transfer_learning"
use_cuda = false
tb_logdir = "tb_TL"

[parameters]

num_epochs = 10
save_every_n_epochs = 5
```

```
batch_size = 50
num_refs = 50
sample_batch_size = 50

input_model_file = "models/reinvent.prior"
smiles_file = "AID_1798002_actives.smi"
output_model_file = "AID_1798002_actives.model"
validation_smiles_file = "AID_1798002_actives.smi"
```

3 Supported Chemistry

The listing S10 summarizes the supported chemistry expressed as tokens for each generator. Basically, all priors support the same elements. The main differences are the ring sizes and that Mol2Mol accepts and generates chiral centers at C and (quaternary) N. Mol2Mol also supports double bond isomers.

Listing S10: Supported tokens in the prior models. Slight variations between models are owing to the specific data set preparation. Special tokens for sequence start, end and padding have been left out. $*$ and | denote attachment points and fragment separator for Libinvent and Linkinvent and are also used in input (see Listings S3 and S4).

```

Reinvent
#, =, -, (, ), 1, 2, 3, 4, 5, 6, 7, 8, 9, %10, Br, C, Cl, F, N, O, S,
    [N+], [N-], [O-], [S+], [n+], [nH], c, n, o, s

Libinvent
decorator:
#, =, -, (, ), 1, 2, 3, 4, 5, 6, Br, C, Cl, F, N, O, S, [N+], [N-],
    [N], [O-], [O], [S+], [n+], [nH], [s+], c, n, o, s, *, |
scaffold:
#, =, -, (, ), 1, 2, 3, 4, 5, 6, 7, 8, 9, Br, C, Cl, F, N, O, S, [*],
    [N+], [N-], [N], [O-], [O], [S+], [n+], [nH], [s+], c, n, o, s

Linkinvent
warheads:
#, =, -, (, ), 1, 2, 3, 4, 5, 6, Br, C, Cl, F, N, O, S, [N+], [O-],
    [O], [S+], [n+], [nH], [s+], c, n, o, s, *, |
linker:
#, =, -, (, ), 1, 2, 3, 4, 5, 6, 7, Br, C, Cl, F, N, O, S, [*], [N+],
    [N-], [O-], [S+], [n+], [nH], [s+], c, n, o, s

Mol2mol (high, medium, low similarities)
```

```
#, =, -, /, \, (, ), 1, 2, 3, 4, 5, 6, 7, 8, Br, C, Cl, F, I, N, O, S, [C@@H], [C@@], [C@H], [C@], [N+], [N@+], [N@@+], [N@], [O-], [O], [S@@], [S@], [n+], [nH], c, n, o, s

Mol2mol mmp:
#, =, -, /, \, (, ), 1, 2, 3, 4, 5, 6, 7, 8, Br, C, Cl, F, I, N, O, S, [C@@H], [C@@], [C@H], [C@], [N+], [N@+], [N@@+], [O-], [O], [S@@], [S@], [n+], [nH], c, n, o, s

Mol2mol scaffold:
#, =, -, /, \, (, ), 1, 2, 3, 4, 5, 6, 7, 8, Br, C, Cl, F, I, N, O, S, [C@@H], [C@@], [C@H], [C@], [N+], [N@+], [N@@+], [O-], [O], [S@@], [S@], [n+], [nH], c, n, o, s

Mol2mol scaffold_generic:
#, =, -, /, \, (, ), 1, 2, 3, 4, 5, 6, 7, 8, Br, C, Cl, F, I, N, O, S, [C@@H], [C@@], [C@H], [C@], [N+], [N@+], [N@@+], [O-], [O], [S@@], [S@], [n+], [n-], [nH], c, n, o, s
```
